# Clustering drug-drug interaction networks with energy model layouts: community analysis and drug repurposing

**DOI:** 10.1038/srep32745

**Published:** 2016-09-07

**Authors:** Lucreţia Udrescu, Laura Sbârcea, Alexandru Topîrceanu, Alexandru Iovanovici, Ludovic Kurunczi, Paul Bogdan, Mihai Udrescu

**Affiliations:** 1 “Victor Babeş” University of Medicine and Pharmacy Timişoara, Faculty of Pharmacy, Timişoara, 300041, Romania; 2 University Politehnica of Timişoara, Department of Computer and Information Technology, Timişoara, 300223, Romania; 3 Institute of Chemistry Timişoara of the Romanian Academy, Timişoara, 300223, Romania; 4 University of Southern California, Ming Hsieh Department of Electrical Engineering, Los Angeles, CA 90089-2563, USA

## Abstract

Analyzing drug-drug interactions may unravel previously unknown drug action patterns, leading to the development of new drug discovery tools. We present a new approach to analyzing drug-drug interaction networks, based on clustering and topological community detection techniques that are specific to complex network science. Our methodology uncovers functional drug categories along with the intricate relationships between them. Using modularity-based and energy-model layout community detection algorithms, we link the network clusters to 9 relevant pharmacological properties. Out of the 1141 drugs from the DrugBank 4.1 database, our extensive literature survey and cross-checking with other databases such as Drugs.com, RxList, and DrugBank 4.3 confirm the predicted properties for 85% of the drugs. As such, we argue that network analysis offers a high-level grasp on a wide area of pharmacological aspects, indicating possible unaccounted interactions and missing pharmacological properties that can lead to drug repositioning for the 15% drugs which seem to be inconsistent with the predicted property. Also, by using network centralities, we can rank drugs according to their interaction potential for both simple and complex multi-pathology therapies. Moreover, our clustering approach can be extended for applications such as analyzing drug-target interactions or phenotyping patients in personalized medicine applications.

Drug repositioning or repurposing is an emerging concept that consists of identifying new therapeutic indications for already existing active pharmaceutical ingredients^1^. Over the recent years, repositioning strategies have been intensely investigated, due to the outstanding advances in scientific and technological fields^2^,^3^. The motivation behind this trend is the fact that, despite the constantly growing resources invested in drug discovery^4^, the drug design process is still cumbersome, slow and prone to many errors^5^,^6^. As a result, the number of new approved bio-active molecules is not increasing anymore^7^; therefore, the pharmaceutical industry is forced to come up with alternative solutions^8^. The fact that the repurposing strategy can be the right answer for current challenges in the pharmaceutical industry is further stressed by a recent report, which states that 20% of the new drugs brought on the market in 2013 are actually repositionings^9^. Another motivation for drug repositioning is that it fits the aims and scopes of personalized and precision medicine^10^.

Traditionally, drug repositioning mostly relies on chance and it is achieved by experimentally exploring the link between molecular structure and biological activity^11^. The advent of big data gathering and analysis has spurred the use of computational approaches in many aspects of pharmacology and drug design, including drug repurposing. Indeed, computational models are used to uncover drug interactions which were not discovered during clinical trials^12^, or to predict drug safety^13^. Moreover, using in-silico tools creates a visual and intuitive system for representing drug interactions^14^, thus helping medical and pharmaceutical practice. In the case of drug repositioning, computational strategies explore the relationships between drug databases on one hand, and genomic, transcriptomic and phenotypic data on the other hand^2^. The computational approaches used to perform the exploration of correlations between the large amounts of genomic, phenotypic and chemical data are data mining, machine learning and network analysis. All rendered repositioning solutions are validated by experimental methods (*in vivo* and *in vitro*) or by automated, computer-based searches in health databases^2^.

Capitalizing on the continuously increasing volume of drug interaction data, as well as on the recent advances in the field of network science, translational pharmacology uses the so-called *drug-drug interactomes* (DDI). A DDI is a complex network in which the nodes represent drugs and the links between them correspond to drug interaction relationships such as common mediation by a specific enzyme. The benefits of processing DDIs with network analysis are threefold. First, the researchers can predict potential interactions that were previously unknown^12^,^15^; this idea is behind the development of software tools for drug interaction alert^16^. Second, the computer-aided analysis of DDIs can assure, right from the drug design process, that certain interactions will be avoided^17^,^18^. Third, DDIs can be used to explore the relationships which link the pharmaceutical properties to drug interactions. Most such previous approaches start with already known pharmaceutical properties in order to predict drug-drug interactions^19^,^20^. However, recent research suggests that interaction information from DDI alone can be used in order to predict physiological drug effects and, consequently, to perform drug repositioning^21^. For instance, in ref. 22, the authors analyze the DDI drugs with Markov Clustering Algorithm, obtaining drug categories that are correlated with some drug functions. Another recent approach uses social media in order to keep track of adverse drug effects as they are reflected by social interaction and, subsequently, to build the DDI that suggests possible repositionings^23^.

## Results

**Figure 1:**
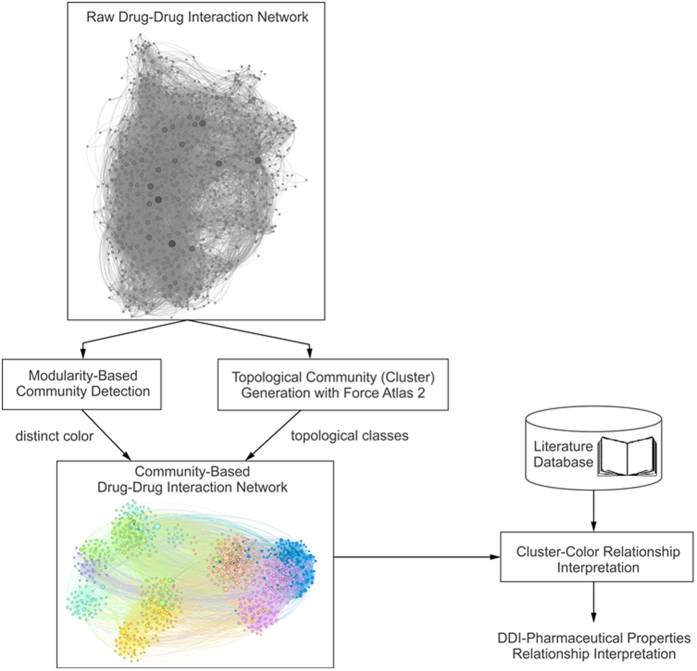
The drug-drug interactome (DDI) analysis procedure for clustering drugs according to relevant pharmacological properties. Our processing procedure is based on modularity classes and energy model topological clustering (Force Atlas 2).

We take the drug-drug interaction data from DrugBank 4.1^24^ (each individual drug has a corresponding list of drug interactions) to build a raw DDI in Gephi^25^, as presented on the top of Fig. 1. Each node corresponds to a distinct drug; a link between two drugs corresponds to an interaction between them according to the considered database. No information regarding the structural or functional properties of these drugs is used throughout the process. We opt not to discriminate between the types of drug interactions (i.e. synergistic or antagonistic), because any kind of interaction contributes to defining the functional profile of a drug category or cluster. Also, we use the older DrugBank 4.1 to build our DDI, so that a later version (DrugBank 4.3) can be used to validate the newly found pharmacological properties in a fair manner.

**Figure 2:**
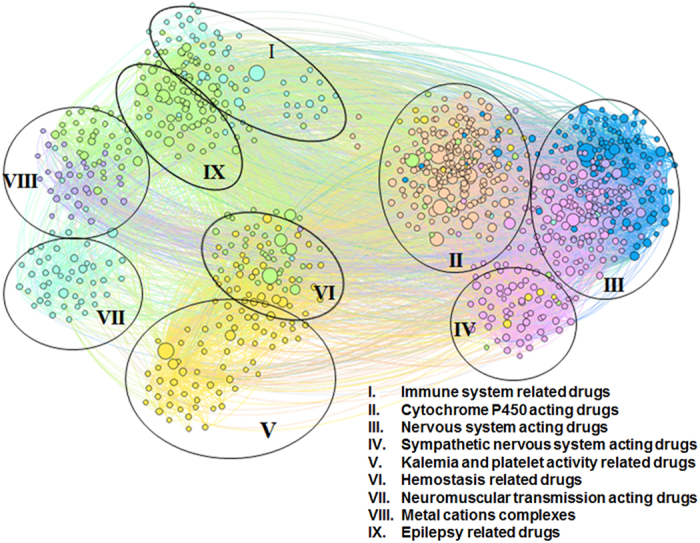
Community-based drug-drug interaction network (CBDDIN) generated in Gephi with interaction data from DrugBank 4.1, containing 1141 nodes (representing drugs) and 11688 links (representing drug-drug interactions). Topological clusters and functional communities are highlighted by using the Force Atlas 2 layout and color labeling of modularity classes.

**Figure 3:**
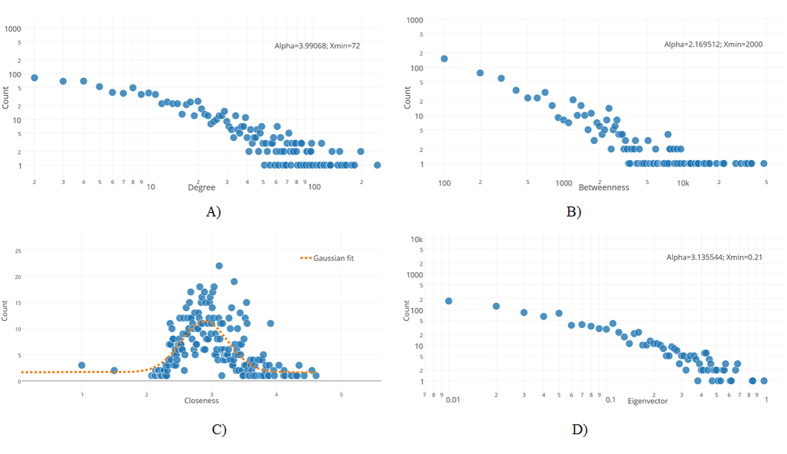
Centrality metrics for the community-based drug-drug interaction network (CBDDIN): (**A**) degree distribution (**B**) betweenness distribution (**C**) closeness distribution, and (**D**) eigenvector distribution. The power law parameters, slope *α* and cutoff point *X*_min_, are provided for degree, betwenness and eigenvector distributions; for the closeness distribution, the best fit is Gaussian function 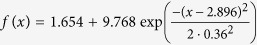 as indicated in panel C).

Then, we apply the procedure suggested in Fig. 1 to automatically assign distinct colors to distinct node modularity classes^26^ and to generate topological clusters (or communities) with energy-model layout algorithm Force Atlas 2. As a result of applying the procedure from Fig. 1, we generate our DDI: the Community Based Drug-Drug Interaction Network (CBDDIN) from Fig. 2. The tools that generate CBDDIN are normally employed in social network analysis^26^,^27^, and the fact that we use them for DDIs is motivated by the topological similarity between our DDI and typical social networks. Indeed, the network statistics for CBDDIN are similar to typical social network statistics: small average path length *L* = 2.978 for a network diameter *Dmt* of 7, significant clustering coefficient *C* = 0.2, average degree 〈*k*〉 = 20.031, modularity *Mod* = 0.452 and density *Dst* = 0.017. Also, Fig. 3 provides the main network centrality distributions, which indicate a scale-free network due to the power-law degree distribution from Fig. 3A. As presented in Fig. 3B,D, betweenness and eigenvector distributions are power-law, while Fig. 3C shows a normal closeness distribution.

Overall, when using statistical fidelity *φ* to measure the topological differences between networks, regardless of their size^28^, we obtain very good similarities with typical social networks: *φ* = 0.780 between CBDDIN and both Facebook and Twitter, and 0.740 between CBDDIN and G^+^.

### Network centrality analysis

The structure of CBDDIN is dictated by drug interaction relationships. Therefore, drugs that are the most prone to drug-drug interactions correspond to the CBDDIN nodes with the biggest network centrality values. Table 1 presents the top 10 drugs, in terms of drug-drug interaction centrality, based on degree, betweenness, closeness, page rank, and eigenvector. Analyzing degree and betweenness centrality distributions contributes to better assessing the interaction potential of each individual drug. Our Supplementary Information (section 1, Fig. 1) presents a visual representation of CBDDIN’s node degree and betweenness.

#### Interpretation of drug communities

Topological communities highlighted in Fig. 2 are generated by Force Atlas 2^27^, whereas modularity classes (identified with distinct colors) are automatedly generated according to Girvan and Newman’s algorithm^29^. Both modularity classes and topological communities are labeled according to a common pharmacological property which characterizes the largest majority of drugs within a distinct community or class.

We assign a pharmacological property to the segregated topological community or modularity class according to DrugBank’s terminology, even if we do not use these properties for clusterization. In some cases the property label refers to an organ/system, in other cases to a medical indication, and in other cases to a chemical structure. However, we consider that we should not put functional restrictions to our annotation process; instead, we look only for the general property that identifies the largest number of drugs within the modularity class and topological community. Indeed, there are several drug categories within each modularity class and topological community (as respectively presented in Tables 1 and 2 from the Supplementary Information file), but we generalized the included drug categories according to DrugBank terminology, referring to either pharmacodynamic or pharmacokinetic properties (see column *Generalized label* in Tables 1 and 2 from the Supplementary Information). If we consider smaller or larger distinct drug categories, then we will not have a unique label for each community or class.

The modularity-based labeling, as presented in Table 2, is very consistent with the allocated tag and its corresponding interpretation. This result is a consequence of modularity being a very good predictor of properties and functionality^30^. Also, modularity is directly linked to the distribution and density of links, which in our case represent drug interactions.

The accuracy of pharmacological labeling of the nine topological communities is reported, on average, in Table 3. Each community has nodes with one or several modularity classes which confirm the topological community tag. The accuracy percentage of color confirmation for the topological communities is provided in the fourth column (Conf. by mod. [%]). Although the initial confirmation percentages according to properties given in DrugBank 4.1 are not particularly high (i.e. the fourth column in Table 3), we are able to explain the allocated topological community labels by cross-validating with extensive literature survey (see supporting information provided as *SupplementaryCBDDIN.xls* file, tab *Cross-checking references*) and searching other databases, such as Drugs.com^31^, RxList^32^, as well as a later DrugBank version (i.e. DrugBank 4.3^24^, last accessed April 2016.). The summarizing result of this cross-validating process is presented in the fifth column of Table 3, which contains the percentages of drug labels which are further confirmed. According to Noack^26^, force-directed energy layouts such as Force Atlas 2 are producing the same clustering as modularity but, at the same time, they contain additional information regarding the nodes’ positions (i.e. if nodes are central or eccentric within the topological cluster, if a node is at the border between topological clusters, etc.)

Taken together, the results show that, although the network is generated based on drug-drug interaction information only, an average of 63% drug labels are found to be in agreement with the properties listed in DrugBank 4.1, whereas 22% are not listed but are subsequently confirmed by an extensive cross-checking process. Thus, a low percentage of drugs (15%) seem to be out of place in their respective topological communities. As such, we can deliver repositioning hypotheses by considering these cases of drugs that seem not to comply with either topological or modularity labels; it is the case of drugs from column “Not expl. [%]” (not explained) in Table 3. Indeed, we suggest that the “not explained” drugs can be repositioned to a property that corresponds to either their modularity class or their topological community. Another repurposing strategy is to consider drugs that topologically lie at the border between two communities; this situation indicate that such drugs may have both pharmacological properties of the neighboring communities.

**Community I: Immune system related drugs.** This community contains antineoplastic agents, immunostimulants and immunosuppressants. In this community, the nodes’ LB color indicates antineoplastic and immunomodulatory properties. The presence of only one VM node in this community (busulfan) is based on its antineoplastic activity, besides being a substrate for cytochrome P450 3A4 (CYP3A4)^24^.

**Community II: CYP P450 acting drugs.** The community consists of substrates, inhibitors and inducers of specific cytochrome P450 enzymes and it includes VM, GB, G, DB, LB and M nodes. VM is directly related to CYP; however, the presence of different color nodes is justified by the fact that, although being characterized by other properties, they also have a CYP-related activity. For instance, oxaliplatin lays within community II because it is identified as a strong CYP2E1 inducer. Oxaliplatin is an antineoplatic agent–this property is indicated by the LB modularity class^24^.

**Community III: Nervous system acting drugs.** This cluster includes drugs that interfere with the metabolism of all neurotransmitters, inducing central as well as peripheral nervous effects. The node inspection reveals the presence of DB, M, LB, and GB colors. DB and M are directly related to the nervous system, while GB and LB correspond to drugs that have other primary properties but generate additional nervous system effects. One such example is guanabenz, a centrally acting antihypertensive that also pertains to modularity class LB because it inhibits 5-lipoxygenase, thus having anti-inflammatory effect^33^.

**Community IV: Sympathetic nervous system acting drugs.** Here we have the classes of drugs that directly and indirectly act on alpha- and beta- adrenoreceptors, including drugs mimicking or inhibiting the sympathetic nervous system (SNS) effects. The included node colors are M (the large majority), GB, G, and DB, because M is directly linked to SNS, and GB, G, DB are additionally acting on SNS (e.g. tolbutamide is placed in community IV because it augments the vasoconstrictor effect of catecholamines^34^,^35^ and has GB modularity due to its anti-platelet aggregation activity^36^,^37^).

**Community V: Kalemia and platelet activity related drugs.** The majority of this community consists of renin-angiotensin system acting drugs and diuretics–with confirmed influence on kalemia and platelet activity–, as well as drugs that are used as platelet aggregation inhibitors. Because all nodes are GB, the community is described as platelet activity and plasma potassium level related drugs, thus confirming the strong connection between the serum potassium concentration and the platelet reactivity^38^.

**Community VI: Hemostasis related drugs.** There are two characteristic node colors, namely GB and G, both of them characterizing drugs that interfere in different phases of hemostasis. Other nodes are LB, M, and P; the presence of LB (pentoxifylline) and P (levothyroxine) nodes is justified by their properties related to hemostasis (both are platelet aggregation inhibitors). Apparently, the presence of the M node (exenatide) within this community has no explanation.

**Community VII: Neuromuscular transmission acting drugs.** All nodes have the same LB color, therefore the community mainly consists of drugs with neuromuscular-blocking activity, as a pharmacodynamic effect, as well as a pharmacotoxicologic effect. However, the community also contains immunosuppressive drugs (e.g. azathioprine) that are used in the treatment of myasthenia gravis (an autoimmune disease affecting the neuromuscular junction^39^,^40^). The same dominant modularity class nodes (labeled as LB) can be found in Community I; this observation is explained by the fact that modularity class LB corresponds to drugs that act on the immune system.

**Community VIII: Metal cations complexes.** The community is generally characterized by two colors, P and G. Bi- and trivalent metal cations are represented by some P nodes. At the same time, all G nodes and the other P nodes are the corresponding chelators. Most drugs (i.e. tetracycline antibiotics, biphosphonates) form unabsorbable complexes with cations such as Ca^2+^, Mg^2+^, Al^3+^ or other polyvalent cation^24^. Other drugs (i.e. ampicillin, amoxicillin, penicillins V and G) degrade under the catalytic influence of transition metal cations^41^. Two GB nodes are placed here because they are metal chelators: deferasirox for iron and penicillamine for copper^24^; their GB modularity class mirrors the platelet aggregation inhibitor effect^42^,^43^.

**Community IX: Epilepsy related drugs.** A portion of nodes colored with G within this community represent drugs which are used for the treatment of various epilepsy and seizure types; the other portion represents epileptogenic drugs. There are also five LB nodes, due to their direct relation with seizure manifestation; for instance, the neurotoxicity of anti-cancer drug cisplatin consists of epileptic seizures^44^,^45^.

### Illustrating examples

In order to demonstrate that our methodology is able to recover multiple pharmacological properties as well as known repositionings, using only information about drug-drug interactions, we present the following cases.

**Figure 4:**
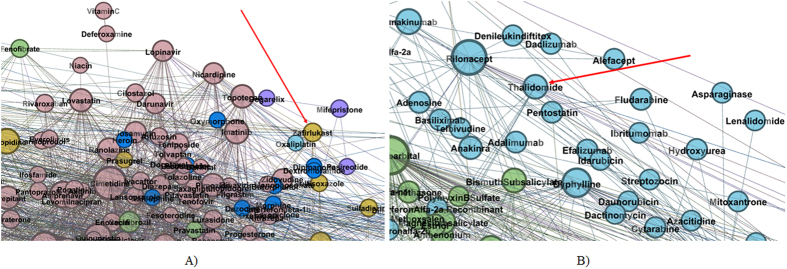
Zoomed details of our DDI indicating. (**A**) Zafirlukast placement within topological community II (**B**) Thalidomide placement within topological community I.

- Zafirlukast is an example for recovering multiple already known pharmacological properties. As such, zafirlukast is an oral leukotriene receptor antagonist used in asthma therapy. Its placement in topological community II (see Fig. 4A) is based on its CYP3A4 activity (substrate and inhibitor) according to DrugBank 4.1^24^ and RxList^32^. Unlike most other nodes within community II, zafirlukast is golden brown (GB), because it interferes with platelet activity by inhibiting the platelet-activating factor^46^.
- Thalidomide is a well-known example of successful drug repositioning; this drug was first used for preventing morning sickness in pregnant women, but then withdrawn because of its teratogenic effects. Nowadays, thalidomide is used in immunological and inflammatory diseases^47^. Indeed, as Fig. 4B presents, thalidomide is confirmed by our method as having anti-cancerous activity because it is a light blue (LB) node placed in Community I (Immune system related drugs).

Our Supplementary Information’s section 3 presents other illustrative examples, which are structured as: examples for recovering multiple well-known drug properties, examples for reconstructing some known drug repositionings, and two lists of possible new drug properties (Tables 3 and 4 in Supplementary Information), which can be developed as new drug repurposings or as new drug interaction discoveries.

## Discussion

Our CBDDIN is characterizing drug’s interaction potential for therapies with a small number of associated pathologies. On the other hand, betweenness and closeness have a non-local character, making them appropriate in characterizing drug interactions in the case of therapies for polypathologies. Furthemore, due to the fact that degree and betweenness are power-law distributed, we are able to identify the drugs with the highest potential for drug-drug interactions (see Table 1).

By using our dual clustering methodology for drug-drug interaction network community analysis, we identify 9 pharmacological characteristics (pointed by the community labels in Fig. 2); this may suggest that the 9 characteristics are paramount in determining and interpreting drug-drug interactions. Unveiling that certain pharmacological properties are more important than others in terms of drug-drug interactions is a clear contribution to the state-of-the-art.

The effectiveness of clustering CBDDIN drug communities according to specific pharmacological properties is confirmed for 85% of drugs from DrugBank 4.1., by cross-checking with other drug databases and extensive literature survey. Consequently, this high prediction accuracy indicates that, for the remaining 15% drugs, there is a high probability that the pharmacological properties predicted by our dual clustering methodology will be confirmed. We prefer the general term “property” because, according to the topological community or modularity class label, the property can be of pharmacokinetic, pharmacodynamic or pharmacotoxicological nature; as such, confirming a predicted property can lead to discovering a new interaction, a new indication (repositioning) or a new possible side-effect, respectively. As possible repositioning examples, our method predicts that chlorzoxazone has immunological properties because it is placed in topological community I. Cefalosporins such as cefalotin, cefamandole, cefixime, etc. also present certain effects on the immune system or neuromuscular junction, as they are placed in community VII. As possible new interaction examples are bevacizumab or lorazepam which possible interfere with CYP enzymes, due to their positioning within community II. The details for all 15% drugs that have unexplained modularity classes and positions within the respective communities, leading to potential new properties, are provided in section 3.3 of our Supplementary Information file (Tables 3 and 4). We suggest that these drugs be further investigated with *in vivo* and *in vitro* techniques, in order to confirm possible repositionings or new interactions.

We observe that topological communities IX (Epilepsy related drugs) and I (Immune system related drugs) present a significant overlapping zone (see Figures 12 and 13 in Supplementary Information’s section 3.2); this overlapping suggests that there is a connection between the two drug categories, namely epilepsy-related and immune system acting. As a confirmation, recent research results indicate disulfiram as an anticancer drug^48^. Indeed, in our CBDDIN the epileptogenic disulfiram is placed in Community IX–Community I overlapping zone (Supplementary Information, Figure 12). Moreover, medroxyprogesterone and megestrol are also placed in Community IX–Community I overlapping zone (Supplementary Information, Figure 11), because they have anti-epileptic activity^49^,^50^ and, at the same time, are hormonal antineoplastic agents^24^. The connection between antineoplastic endocrine drugs and epilepsy is further strengthened by recent research^51^ which reports that anticancer drugs letrozole and fadrozole substantially mitigate seizures.

Drug-drug interactions reflect the interference of drug behaviors. In order to infer possible repositionings, our strategy relies on the behavioral relationships between drugs rather than the structural similarities represented by chemical structure relationships or drug-target relationships. In order to reduce the complexity entailed by drug repurposing, we suggest that our behavioral perspective can be integrated with the complementary structural perspective^52^, which can rely on various sets of data (genome, phenome, drug-target combinations, etc.) Therefore, we indicate the Big Mechanism project^53^ as a possible integrator platform that can gather and process all repurposing information from different perspectives and tools.

Our CBDDIN is not the only bio-medical complex network that is similar to social networks; as such, our methodology is very useful for clustering patients in medical studies based on their compatibility, which is defined in terms of anthropometric measures, questionnaire results and simple clinical data (such as hypertension value, body temperature, oxygen desaturation, etc.) Thus, applications for cardiovascular diseases^54^, apnea^55^, and defining endophenotypes^56^ are using a similar dual clustering methodology in order to show that the multiple disease factors do not associate at random; instead, they converge towards defining patient phenotypes which are useful for designing personalized therapies.

## Methods

### Databases

We build our CBDDIN by using drug interaction information from the public drug database DrugBank Version 4.1^24^, which has 7739 drug entries (among them there are FDA drugs and biotech drugs, nutraceuticals, and experimental drugs.) We filter all the drugs which have no drug-drug interaction information, obtaining 1141 drugs. The verification of interpretations and repositioning predictions obtained by our network-based methodology is performed by cross-checking functional properties in other databases: *Drugs.com, RxList*, and *DrugBank 4.3*. In addition, the cross-checking verification of functional drug properties is also made by searching the literature in electronic format. The list of 309 retrieved papers which confirm the predicted drug properties is given as supporting information (tab *Cross-checking references* in *SupplementaryCBDDIN.xls*).

### Complex network clustering

The processing of our CBDDIN, including computation of statistics, modularity clustering and graphical layouts, is performed in Gephi^25^, a leading tool in visualization and analysis of large networks. The clustering algorithms that we use in this paper are based on modularity classes^26^ and on the energy-model layout Force Atlas 2^27^.

**Definition 1.** An unweighted network consists of a set of vertices 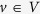 and a set of edges 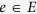 which connect certain pairs of vertices from *V*.

The *average path length L* of a network (*V, E*) is the mean distance between two nodes, averaged over all pairs of nodes; the *clustering coefficient C* is defined as the average fraction of node’s neighbor pairs that are also neighbors to each other^57^. The *degree k* of a node is defined as the total number of its incident edges. Thus, the network’s *degree distribution* is characterized by function *P*(*k*), whereas the *diameter Dmt* is the maximal distance among all distances between any pair of nodes in the (*V, E*) network^57^. The *network density Dst* is defined as the ratio of edges in the network to the total number of possible edges^28^. *Modularity Mod* is a measure that characterizes the strength of dividing the network into communities^29^.

Besides the degree, the main centrality metrics (expressing the importance of a node in the society) are: betweenness, closeness, page rank and eigenvector. The node *betweenness* is defined as the number of minimal paths from all vertices in the network to all other vertices that pass through the node, normalized over the total number of shortest paths. The inverse of the sum of shortest path lengths from one node to all other vertices is called *closeness*. The *eigenvector centrality* computes relative scores for all nodes in the network, by considering that the connections to high-influence vertices are more important than the connections to low-influence vertices; the *page rank* is merely a variant of eigenvector used to rank websites^30^,^58^.

In order to measure the similarity between two complex networks, the network fidelity metric *φ* was introduced^28^:


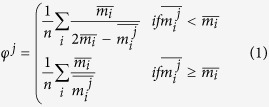


In equation 1, *j* represents the index of the network being compared to the reference network. The index of the network metric which describes the two compared models (e.g. average path length, average degree etc.) is denoted by 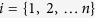, where *n* is the total number of common metrics taken into consideration. Fidelity takes values between 0 and 1 (or as percentiles), with 1 representing perfect similarity. The metric measurements on the reference model are *m*_*i*_, respectively 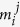 on the model being compared.

**Definition 2.** Given an unweighted network (*V, E*) and an Euclidean *d*-dimensional space 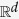, a layout maps each vertex 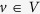 to a position 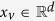 and assigns an Euclidean distance 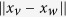 to each edge 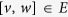.

Energy model layouts are layout algorithms that can be represented as force systems. Many energy model layouts are developed as attraction-repulsion or a-r force systems^26^. In a-r layouts, adjacent vertices attract whereas all other pairs of vertices repulse, thus forming groups of vertices with dense connections (i.e. communities or clusters). The a-r forces are proportional to the power (*a* or *r*) of the Euclidean distances between the nodes: the attraction between adjacent vertices *v* and *w* is 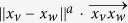 and the repulsion between any two vertices 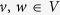 is 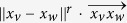 (with 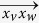 as the unit vector from *v* to *w*). Normally, 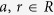, are chosen so that *a* ≥ 0 and *r* ≤ 0, so that attraction is not decreasing and repulsion is not increasing with the Euclidean distance. The most popular force-based layout systems are the model of Fruchterman and Reingold (*a* = 2, *r* = −1)^59^, and the LinLog model (*a* = 0, *r* = −1)^60^.

For all a-r energy models, the resulted layout corresponds to the situation where local energy minimum is attained^26^. As such, total energy for the a-r layout (*a* > *r*) is:


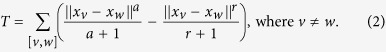


**Definition 3.** Network clustering consists of classifying all the vertices 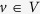 in one of the disjoint vertex subsets (i.e. clusters) *C*, pertaining to the set of disjoint subsets *V*_*C*_^61^.

Several network parameters are used for network clustering in complex networks, but one of the most useful is the modularity which was advocated by Newman and Girvan^29^. In an unweighted network, such as our DDI, the modularity of a clustering *V*_*C*_ is defined as:


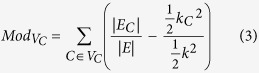


In Equation 3, |*E*_*C*_| is the number of edges in cluster *C*, |*E*| is the total number of edges in the network, *k*_*C*_ is the total degree of nodes in cluster *C*, while *k* is the total degree of nodes in the entire network. At the same time, 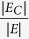 represents the fraction of intra-cluster edge density relative to the density of the entire network (which is assumed to be uniform), while 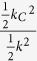 is the expected such fraction. Therefore, modularity grows as clustering produces clusters with edge densities that are larger than expected.

Noack has demonstrated that, when *a* > −1 and *r* > −1, force-directed layout algorithms produce topological clusters which are equivalent with those rendered by modularity-based network clustering^26^. However, force-directed algorithms provide additional topological information about clusters, which leads to recommending the usage of both modularity clustering and a-r force directed layouts for more accurate network analysis^27^.

## Additional Information

**How to cite this article**: Udrescu, L. *et al*. Clustering drug-drug interaction networks with energy model layouts: community analysis and drug repurposing. *Sci. Rep.* **6**, 32745; doi: 10.1038/srep32745 (2016).

## Supplementary Material

Supplementary Information

Supplementary Information

## Figures and Tables

**Table 1:** Community-based drug-drug interaction network (CBDDIN) top 10 drug hierarchies based on degree, betweenness, closeness, page rank, and eigenvector centralities.

	Degree Drug	Value	Betweenness Drug	Value	Closeness Drug	Value	Page rank Drug	Value	Eigenvector Drug	Value
1.	Voriconazole	250	Triprolidine	47.570	Voriconazole	2.077	Triprolidine	0.009	Voriconazole	1
2.	Triprolidine	198	Trastuzumab	37.522	Ketoconazole	2.121	Voriconazole	0.009	Telithromycin	0.851
3.	Telithromycin	198	Treprostinil	37.168	Phenytoin	2.128	Treprostinil	0.008	Trimipramine	0.845
4.	Warfarin	181	Warfarin	34.129	Cyclosporine	2.141	Warfarin	0.008	Rifampin	0.683
5.	Trimipramine	174	Cyclosporine	33.217	Telithromycin	2.142	Trastuzumab	0.007	Tramadol	0.682
6.	Ketoconazole	161	Tacrolimus	30.491	Tacrolimus	2.155	Telithromycin	0.007	Tacrolimus	0.680
7.	Rifampin	157	Tacrine	27.734	Warfarin	2.168	Cyclosporine	0.006	Trazodone	0.656
8.	Cyclosporine	150	Phenytoin	22.018	Rifampin	2.171	Ketoconazole	0.006	Ketoconazole	0.656
9.	Phenytoin	147	Ketoconazole	21.598	Trimipramine	2.180	Tacrine	0.006	Quinidine	0.623
10.	Tacrolimus	147	Rifampin	21.288	Fosphenytoin	2.184	Rifampin	0.006	Clarithromycin	0.609

Values are provided for each centrality metric.

**Table 2:** Interpretation of CBDDIN colors allocated by the modularity clustering algorithm.

Modularity class (color)	Code	Modularity class interpretation	No. of drugs	Consistency [%]
Dark blue	DB	Central and peripheral nervous system acting drugs	232	96
Velvet maroon	VM	Substrates, inhibitors and inducers of specific CYP enzymes	210	91
Green	G	Drugs that interfere in different phases of hemostasis, anticonvulsant and epileptogenic drugs	191	85
Magenta	M	Drugs acting on sympathetic nervous system	166	93
Light blue	LB	Drugs targeting cancer, auto-immune disorders (i.e. rheumatoid arthritis), and musculoskeletal system	156	88
Golden brown	GB	Drugs interfering with platelet activity and plasma potassium levels	155	92
Purple	P	Bi-and trivalent cations, chelating agents	31	100

The tags are allocated to modularity classes according to pharmacological properties (column Modularity class interpretation). The number of drugs pertaining to each modularity class, along with the percentage of drugs that abide by the color interpretation, are also provided (columns No. of drugs and Consistency [%], respectively).

**Table 3:** CBDDIN topological cluster labeling for each of the nine communities.

Comm.	Colors	No.	Conf. by mod. [%]	Expl. by propr. [%]	Not expl. [%]
I	LB, VM	80	77	19	4
II	VM, GB, G, DB, LB, M	271	80	8	12
III	DB, M, LB, GB	307	84	12	4
IV	M, GB, G, DB	81	51	22	27
V	GB	54	33	63	4
VI	GB, G, LB, M, P	125	35	38	27
VII	LB	58	26	31	43
VIII	P, G, LB, GB	69	56	9	35
IX	G, LB	96	30	57	13
		1141	63	22	15

The modularity classes (represented by colors) confirming the community tag are listed in the second column. The third column contains the total number of drugs (No.) for each community (Comm.), which is then apportioned to the following cases: properly described by the assigned modularity class (Conf. by mod.), explained by properties taken from drug databases and literature review (Expl. by propr.), and not explained (Not expl.).
