# Supplementary material for: Clustering drug-drug interaction networks with energy model layouts: community analysis and drug repurposing
Source: Sci Rep. 2016 Sep 7;6:32745. doi: 10.1038/srep32745 (PMC5013446; doi:10.1038/srep32745)
Supplement: Supplementary Information [file srep32745-s1.pdf]

Clustering drug-drug interaction networks with energy model layouts: community analysis and drug repurposing

(Supplementary Information)

Lucreția Udrescu, Laura Sbârcea, Alexandru Topîrceanu, Alexandru Iovanovici,
Ludovic Kurunczi, Paul Bogdan, and Mihai Udrescu

1. Network centrality analysis

The structure of CBDDIN is dictated by drug interaction relationships. Therefore, drugs that are the most prone to drug-drug interactions correspond to CBDDIN nodes with the biggest network centrality values. In Figures 1.a and b, we present CBDDIN with the node size and color intensity being allocated proportionally to node degree and betweenness respectively. We also indicate, with corresponding arrows, the 3 most important drugs in terms of degree and betweenness.

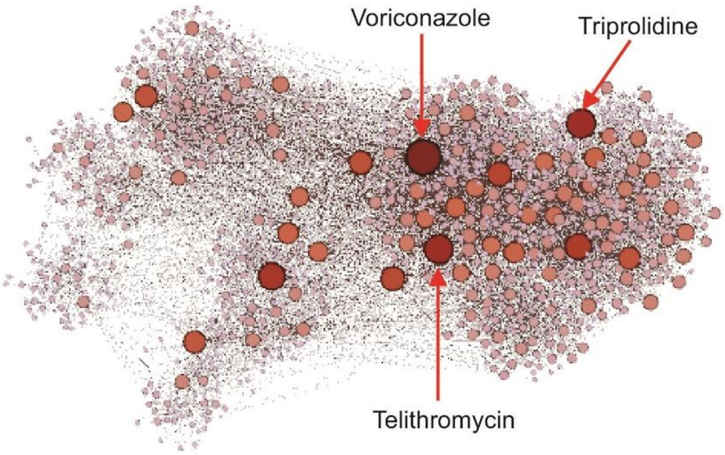

a)

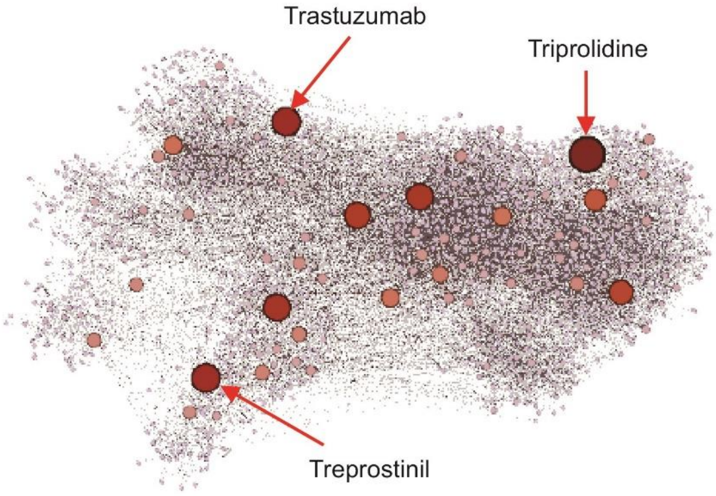

b)

Figure 1. Community-based drug-drug interaction networks which highlight (with node size and color intensity): a) degree distribution, b) betweenness centrality distribution.

2. Explanations on topological communities and modularity classes labeling

Table 1. Explanations for label allocation on modularity classes, represented by colors in Figure 2 from the main manuscript. In the second column (*Drug categories*), we list the drug categories that are present in the corresponding modularity classes. The *Generalized label* in column 3 introduces the actual allocated class label, which generalizes the properties listed as drug categories. The percentage of modularity class drugs which comply with the generalized labels, along with the confirmation references are also listed in columns 4 and 5.

Modularity class (color code)	Drug categories*	Generalized label	Confirmed with the generalized label [%]	Confirmation reference [database or literature]§
Dark blue (DB)	• H1 receptor antagonists for systemic use<br>• Anticholinesterase anti-dementia drugs<br>• Antipsychotics<br>• Antidepressants<br>• Anti-Parkinson drugs<br>• Hypnotics and sedatives<br>• Centrally acting muscle relaxants<br>• Analgesics<br>• Centrally acting sympathomimetic psychostimulants<br>• General anesthetics<br>• Ophthalmological parasympathomimetics and anticholinergics	Central and peripheral nervous system acting drugs	96.69	DB4.1, D, R, L50-L52, L57-L83, L87-L100, L217, L218
Velvet maroon (VM)	• Cytochrome P-450 inhibitors<br>• Cytochrome P-450 inducers<br>• Combined inhibitors of CYP3A4 and P-glycoprotein<br>• Combined inducers of CYP3A4 and P-glycoprotein	Substrates, inhibitors and inducers of specific CYP enzymes	90.95	DB4.1, DB4.3, D, R, L25-L29, L34, L36-39, L43-L46
Green (G)	• Vitamin K antagonists<br>• Vitamin K and derivatives hemostatics<br>• Thyroid hormones<br>• Androgen hormones<br>• Antiepileptics<br>• Barbiturates	Drugs that interfere in different phases of hemostasis, anticonvulsant	85.34	DB4.1, D, R, L69, L179-L188, L192-L193, L205, L224-L249

	• Corticosteroids<br>• Progestogens<br>• Estrogens	and epileptogenic drugs		
Magenta (M)	• Beta-adrenergic antagonists<br>• Alpha- and beta-adrenergic antagonists<br>• Beta-adrenergic bronchodilators<br>• Peripherally acting alpha-adrenergic antagonists<br>• Antiadrenergic antihypertensives<br>• Decongestant alpha-agonists<br>• Ergot alkaloids<br>• Oral antihyperglycemic drugs<br>• Insulins	Drugs acting on sympathetic nervous system	93.37	DB4.1, L250-L263
Light blue (LB)	• Antineoplastic agents<br>• Immunomodulating drugs<br>• Peripherally acting muscle relaxants<br>• Immunosupressants for autoimmune disorders<br>• Uricosuric drugs<br>• Aminoglycoside antibiotics<br>• Lincosamides<br>• Glycopeptide antibiotics	Drugs targeting cancer, autoimmune disorders (i.e. rheumatoid arthritis), and musculoskeletal system	87.82	DB4.1, D, R, L1-L11, L14-L21, L24, L69, L171-L177, L208-L216, L302-L306
Golden brown (GB)	• Renin-angiotensin system acting drugs<br>• Potassium<br>• Diuretics<br>• Platelet aggregation inhibitors<br>• Heparins<br>• NSAIDs<br>• Antithrombotic enzymes	Drugs interfering with plasma potassium level and platelet activity	91.61	DB4.1, DB4.3, D, L138-L140, L143-L144, L148-L155, L158-L167, L264-L290
Purple (P)	• Metal cations<br>• Metal compounds<br>• Biphosphonates<br>• Fluoroquinolones	Bi-and trivalent cations, chelating agents	100.00	DB4.1, DB4.3, D, L307

[*] According to DrugBank terminology

[§] Confirmation references: DB4.1 – DrugBank 4.1, DB4.3 – DrugBank 4.3, D – drugs.com, R – rxlist.com, Li – literature reference from *SupplementaryCBDDIN.xls* file, tab *Cross-checking references*.

Table 2. Explanations for label allocation on topological communities from Figure 2 in the main manuscript. In the second column (*Drug categories*), we list the drug categories that are present in the corresponding topological communities. The *Generalized label* in column 3 introduces the actual allocated community label, which generalizes the properties listed as drug categories. The percentage of topological community drugs which comply with the generalized labels, along with the confirmation references are also listed in columns 4 and 5.

Topological community	Drug categories*	Generalized label	Confirmed with the generalized label [%]	Confirmation reference [database or literature]§
**I**	• Immunosuppressants<br>• Immunostimulants<br>• Monoclonal antibodies<br>• Antineoplastic platinum compounds<br>• Alkylating agents<br>• Antimetabolites<br>• Cytotoxic antibiotics<br>• Protein kinase inhibitors<br>• Adenosine deaminase inhibitors	Immune system related drugs	96.25	DB4.1,<br>L1-L24,<br>L305, L306
**II**	• Cytochrome P-450 inhibitors<br>• Cytochrome P-450 inducers<br>• Combined inhibitors of CYP3A4 and P-glycoprotein<br>• Combined inducers of CYP3A4 and P-glycoprotein	Cytochrome P450 acting drugs	87.45	DB4.1,<br>DB4.3,<br>D, R,<br>L25-L49
**III**	• Hypnotics and sedatives<br>• Anxiolytics<br>• Antipsychotics<br>• Antidepressants<br>• Anti-Parkinson drugs<br>• Anti-dementia drugs<br>• Analgesics<br>• Antiepileptics<br>• Systemic H1 antagonists<br>• General anesthetics<br>• Local anesthetics	Nervous system acting drugs	96.42	DB4.1,<br>D, R,<br>L50-L100
**IV**	• Beta-adrenergic antagonists<br>• Beta-adrenergic agonists<br>• Alpha-adrenergic antagonists<br>• Antiadrenergic antihypertensives	Sympathetic nervous system acting drugs	72.84	DB4.1,<br>DB4.3,<br>D,

	• Organic nitrates<br>• Oral antihyperglycemic drugs<br>• Insulins			L101-L119, L295, L296
**V**	• Renin-angiotensin system acting drugs<br>• Renin inhibitors<br>• Potassium<br>• Diuretics<br>• Platelet aggregation inhibitors<br>• Direct thrombin inhibitors<br>• Heparins	Kalemia and platelet activity related drugs	96.30	DB4.1, L120-LL146, L283, L300
**VI**	• Vitamin K antagonists<br>• Antithrombotic enzymes<br>• Systemic hemostatics<br>• NSAIDs<br>• Thyroid hormones<br>• Androgen hormones	Hemostasis related drugs	73.60	DB4.1, DB4.3, D, L120-L123, L148-L155, L163-170 L219-227, L291-L294
**VII**	• Peripherally acting muscle relaxants<br>• Immunosupressants for autoimmune disorders<br>• Aminoglycoside antibiotics<br>• Lincosamides<br>• Glycopeptide antibiotics	Neuromuscular transmission acting drugs	56.90	DB4.1, D, L69, L171-177, L297-L299
**VIII**	• Metal cations<br>• Metal compounds<br>• Metal chelating drugs<br>• Tetracyclines<br>• Biphosphonates<br>• Fluoroquinolones	Metal cations complexes	65.22	DB4.1, L178, L301, L307-L308
**IX**	• Antiepileptic drugs<br>• Barbiturates<br>• Corticosteroids<br>• Contraceptive progestogens<br>• Antineoplastic progestogens<br>• Contraceptive estrogens<br>• Antineoplastic estrogens	Epilepsy related drugs	87.50	DB4.1, D, R, L179-L207, L304

[*] According to DrugBank terminology

[§] Confirmation references: DB4.1 – DrugBank 4.1, DB4.3 – DrugBank 4.3, D – drugs.com, R – rxlist.com, Li – literature reference from *SupplementaryCBDDIN.xls* file, tab *Cross-checking references*.

3. Illustrating examples

This section presents three types of illustrating examples on our methodology's effectiveness in recovering and predicting pharmacological properties. In section 3.1 we present examples for recovering multiple well-known drug properties. Section 3.2 illustrates the fact that our methodology is capable of reconstructing some known drug repositionings, whereas section 3.3 proposes two lists of possible new drug properties, which can be developed as new drug repurposings or as new drug interaction discoveries.

3.1 Multiple properties

3.1.1 Busulfan

Busulfan is an alkylating antineoplastic drug with immunosuppressive properties, which places it within Community I (Immune system related drugs). Also, busulfan stands out by its velvet maroon (VM) color within Community I (where the majority of nodes are LB), because it is a substrate for CYP3A4 and its pharmacological effect is strongly influenced by the CYP3A4 inhibitors. This interpretation is confirmed by reference [1].

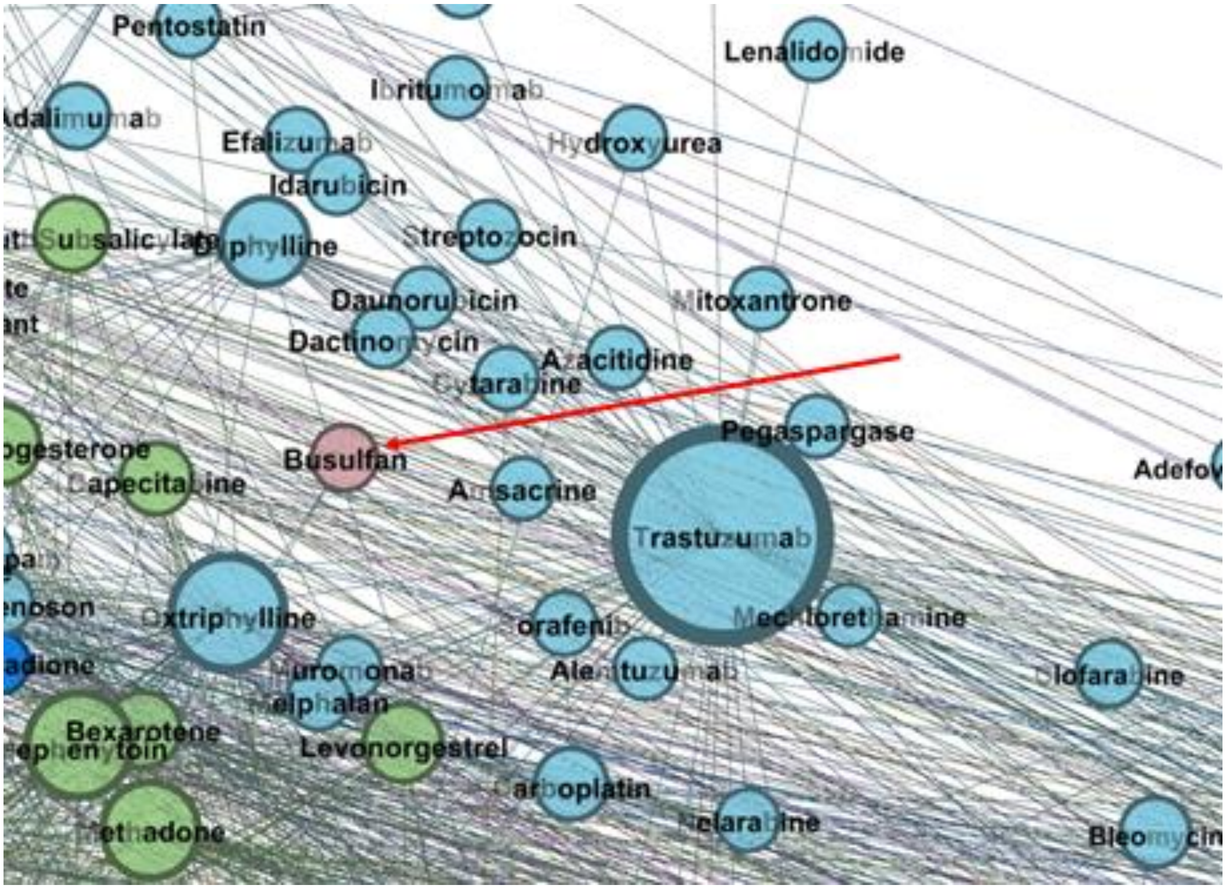

Figure 2. Zoomed busulfan placement within topological Community I (Immune system acting drugs).

3.1.2 Tolbutamide

Tolbutamide is placed in topological Community IV (Sympathetic nervous system acting drugs), which is backed by its property of increasing the constrictor effect of catecholamine, similarly to other oral hypoglycemic agents [2][3]. Although the majority of this topological community's nodes are magenta (M), tolbutamide is colored in golden brown (GB); this is explained by the demonstrated direct antiplatelet activity, independent of the hypoglycemiant effect of tolbutamide, as validated by literature surveys [4][5].

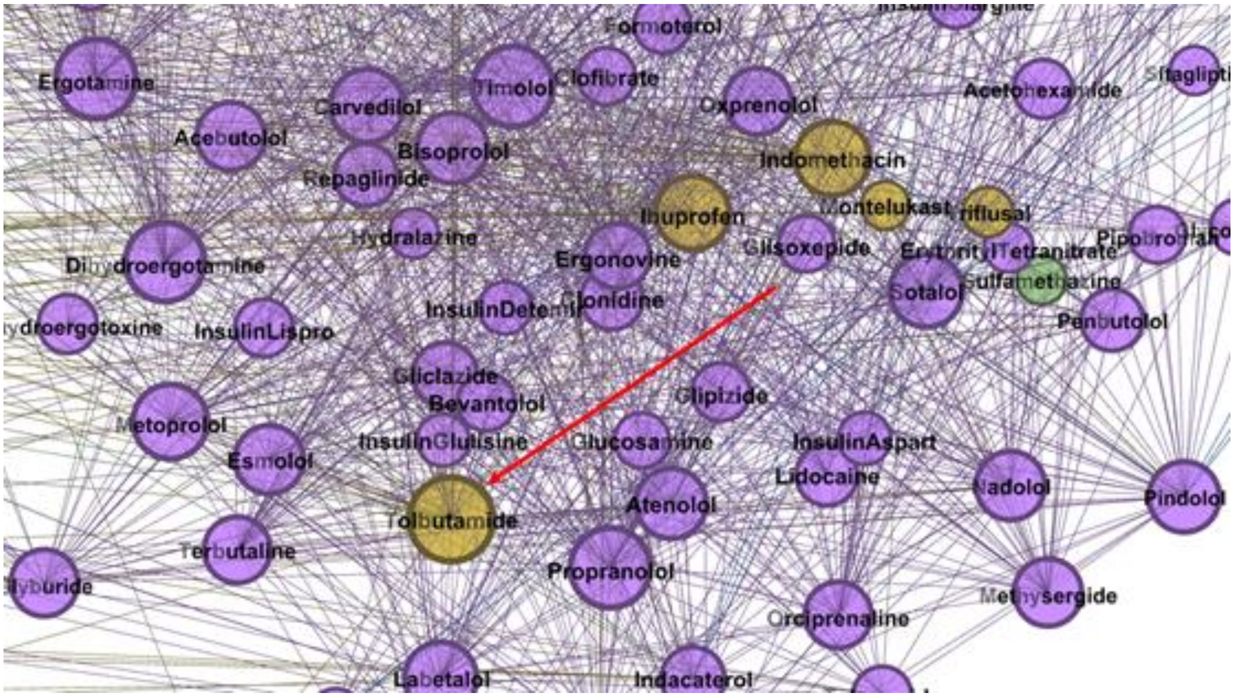

Figure 3. Zoomed tolbutamide placement within topological Community IV (Sympathetic nervous system acting drugs).

3.1.3 Ticlopidine

Ticlopidine is a platelet aggregation inhibitor pertaining to GB (golden brown) modularity class, which consists of drugs interfering with platelet activity. Ticlopidine is placed within topological Community II (Cytochromes P450 substrates/inhibitors/inducers) because CYP3A4, CYP2C19, CYP2D6 are involved in ticlopidine's liver metabolism[1].

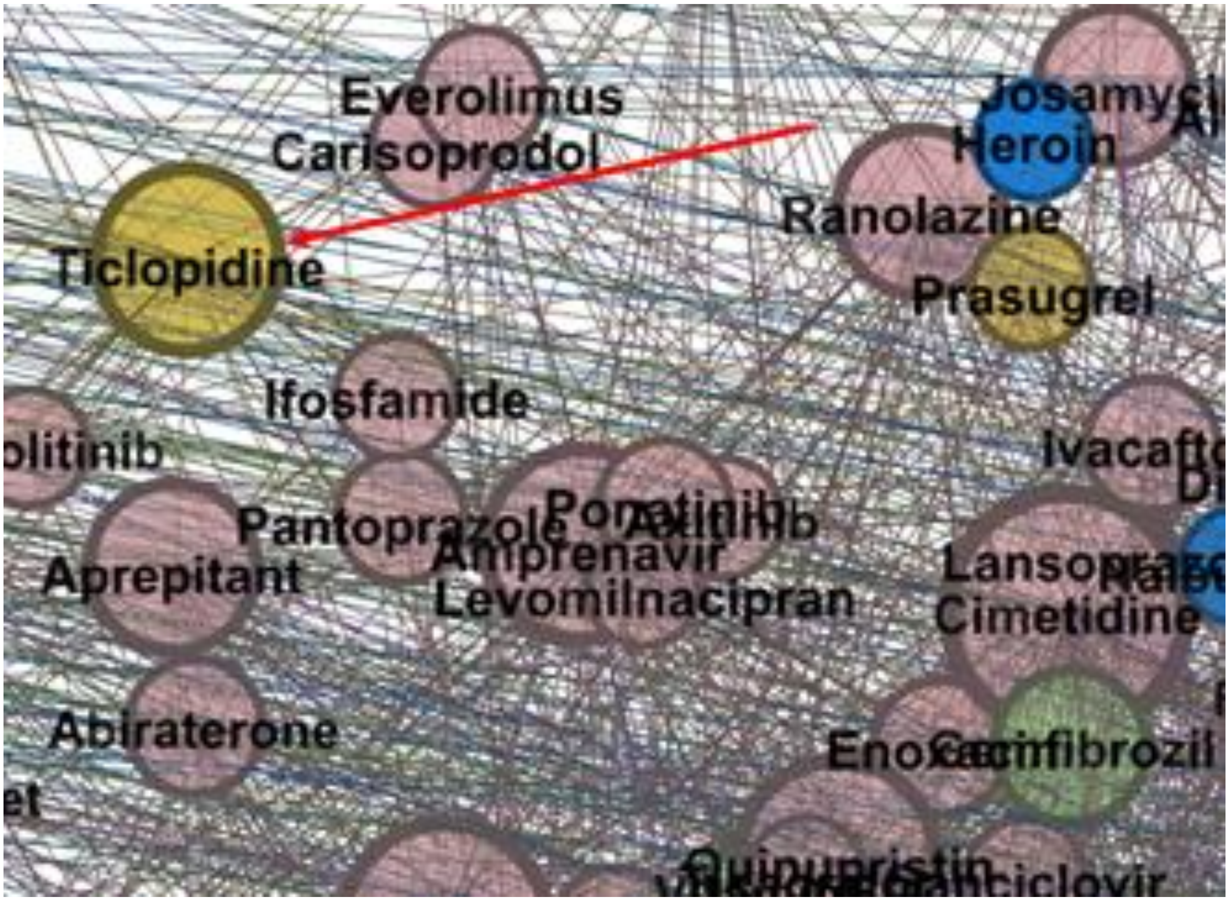

Figure 4. Zoomed ticlopidine within Community II (Cytochromes P450 substrates/inhibitors/ inducers).

[1] Wishart DS, Knox C, Guo AC, Shrivastava S, Hassanali M, Stothard P, Chang Z, Woolsey J. DrugBank: a comprehensive resource for in silico drug discovery and exploration. Nucleic Acids Res. 2006 Jan 1;34(Database issue):D668-72 [http://www.drugbank.ca/]

3.1.4 Oxaliplatin

Oxaliplatin is a LB (light blue) node because of its antineoplastic properties[2]. At the same time, oxaliplatin is placed within topological Community II (CYP enzymes acting drugs), because it interacts with liver cytochromes P450 [6].

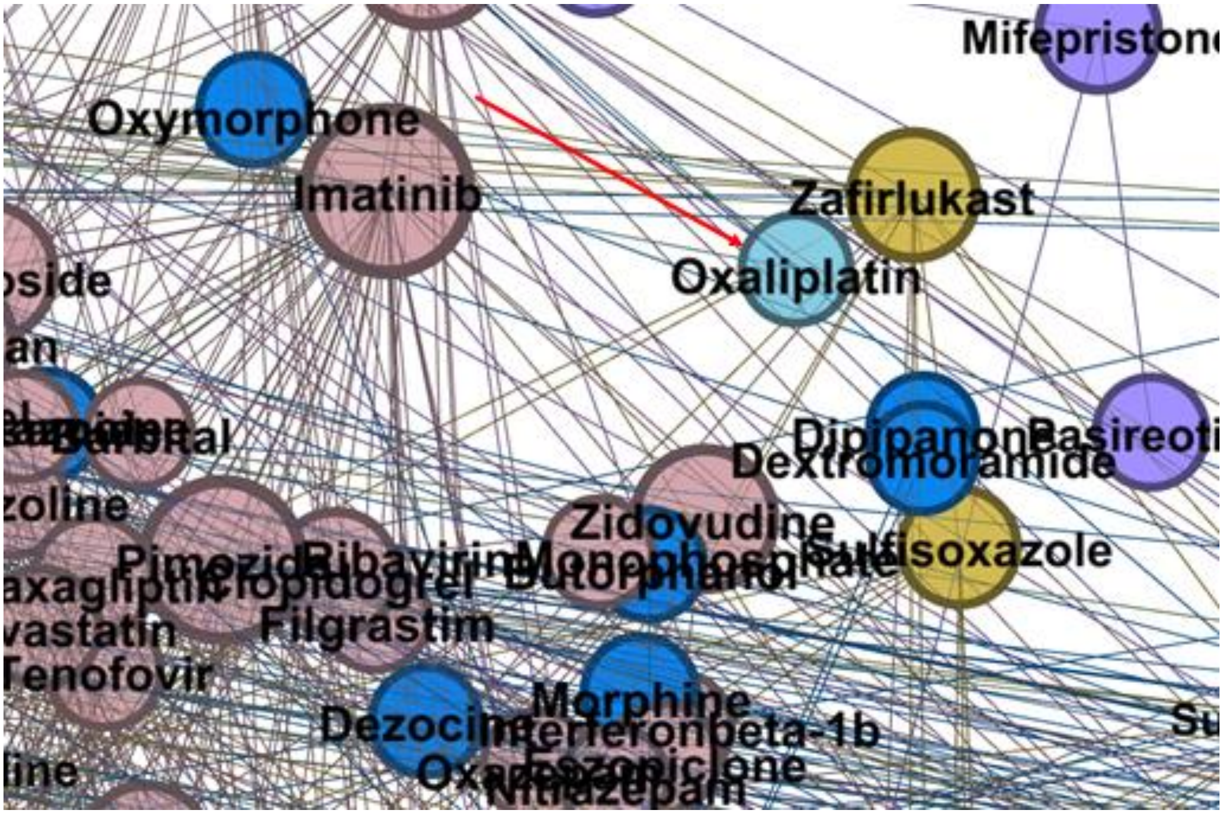

Figure 5. Zoomed oxaliplatin within Community II (Cytochromes P450 acting drugs).

[2] Wishart DS, Knox C, Guo AC, Shrivastava S, Hassanali M, Stothard P, Chang Z, Woolsey J. DrugBank: a comprehensive resource for in silico drug discovery and exploration. Nucleic Acids Res. 2006 Jan 1;34(Database issue):D668-72 [http://www.drugbank.ca/]

3.1.5 Pentoxifylline

Pentoxifylline is a drug which topologically lays within community VI, because it improves blood rheology and inhibits platelet aggregation[3]. However, the light blue (LB) modularity class indicates an immunological property that is confirmed by cross-checking with DrugBank 4.3: pentoxifylline stimulates the production of cytokine, hence modulating immunologic activity[3].

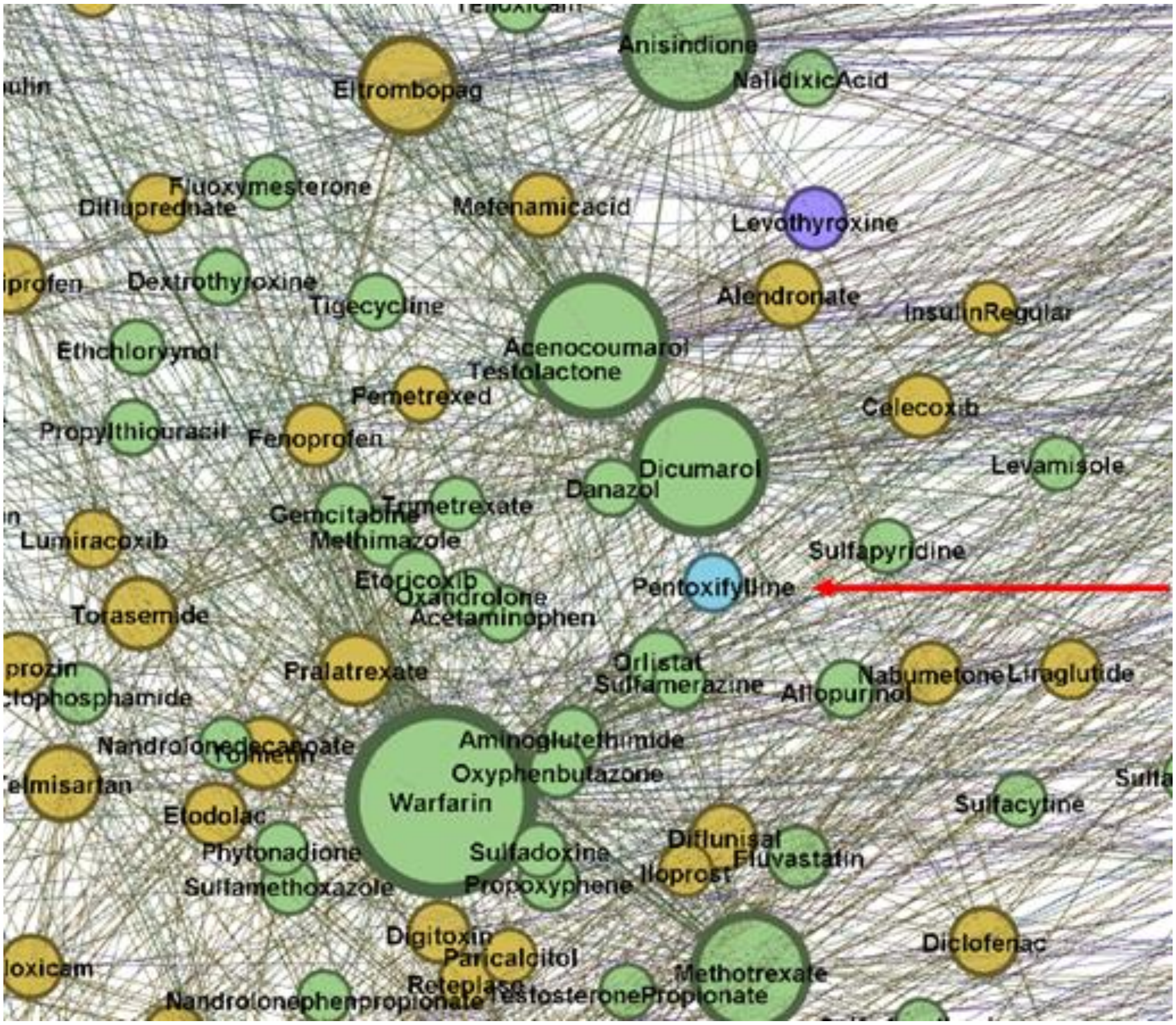

Figure 6. Zoomed pentoxifylline within Community VI (Hemostasis related drugs).

---

[3] Wishart DS, Knox C, Guo AC, Shrivastava S, Hassanali M, Stothard P, Chang Z, Woolsey J. DrugBank: a comprehensive resource for in silico drug discovery and exploration. Nucleic Acids Res. 2006 Jan 1;34(Database issue):D668-72 [http://www.drugbank.ca/]

3.2 Known repositionings

3.2.1 Thalidomide

Thalidomide is a well-known example of successful drug repositioning; this drug was first used for preventing morning sickness in pregnant women, but then withdrawn because of its teratogenic effects. Nowadays, thalidomide is used in immunological and inflammatory diseases [7][8][9][10][11]. Indeed, thalidomide is confirmed by our method as having anti-cancerous activity because it is a light blue (LB) node placed in Community I (Immune system related drugs).

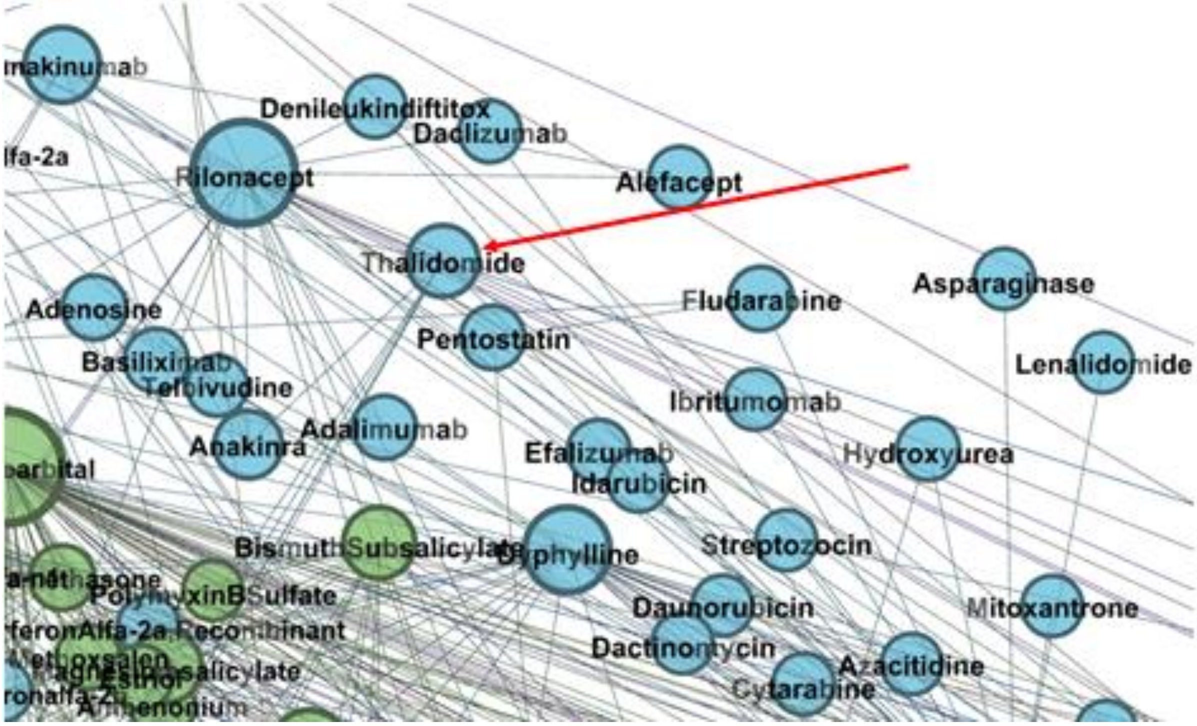

Figure 7. Zoomed thalidomide topological placement within Community I (Immune system acting drugs).

3.2.2 Galantamine

Galantamine (DB node in Community III – Nervous system acting drugs), an anticholinesterase inhibitor which was used to treat myasthenia gravis, is repurposed in Alzheimer’s disease [12][13][14].

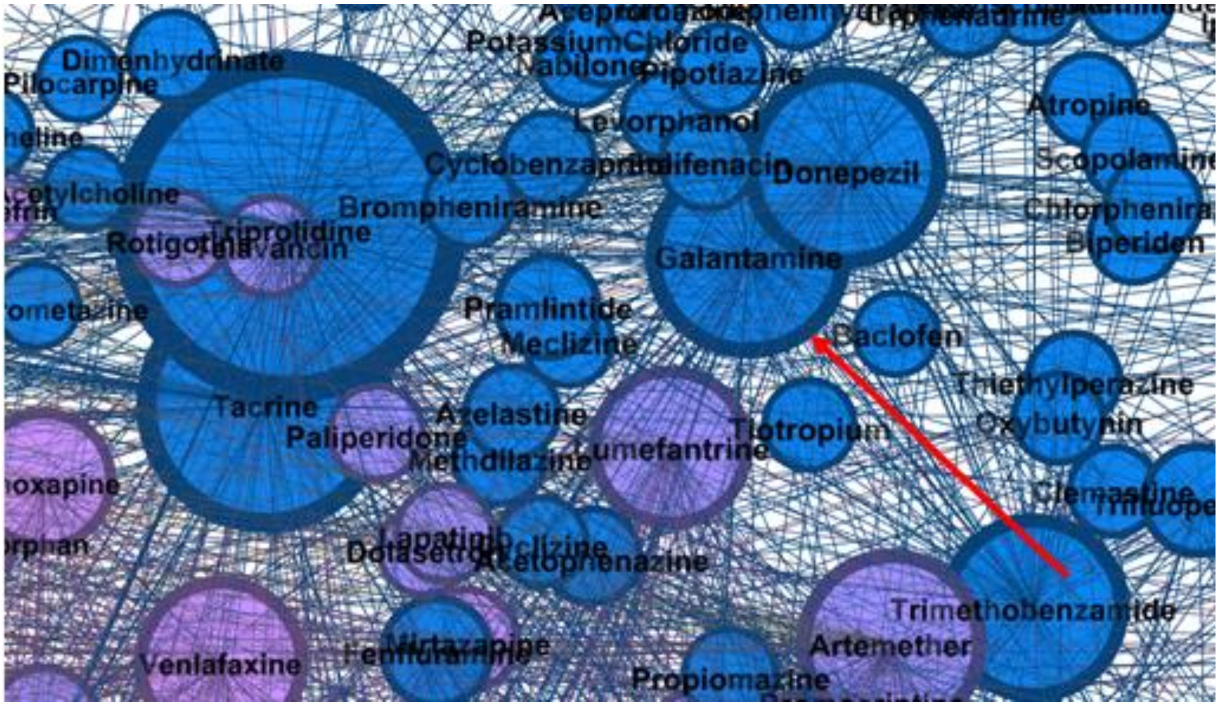

Figure 8. Zoomed galantamine in Community III (Nervous system acting drugs).

3.2.3 *Methylxanthines*

Bronchodilator methylxanthines are LB nodes in Community I (Immune system related drugs) due to their immunomodulatory properties [15][16]. Indeed, there is substantial evidence on the effectiveness of methylxanthines as anticancer drugs [17][18][19].

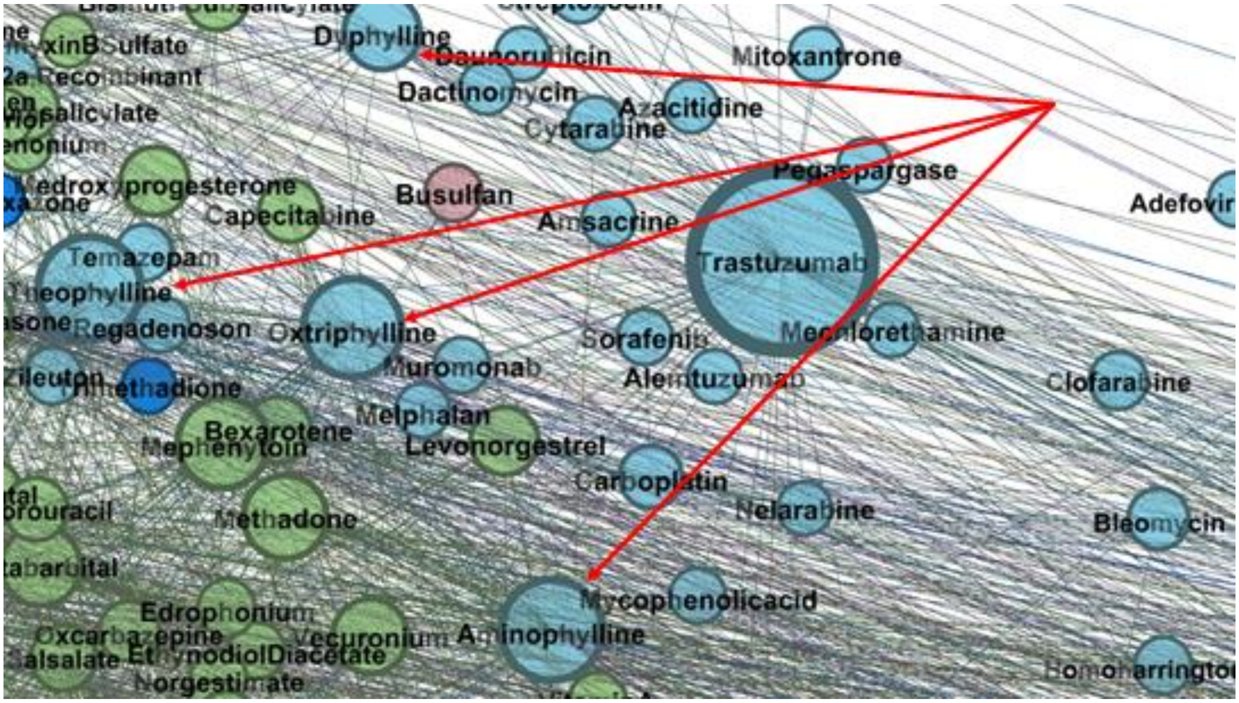

Figure 9. Zoomed theophylline, aminophylline, dyphylline and oxtriphylline positions within Community I (Immune system related drugs).

3.2.4 Zileuton

The leukotriene receptor antagonist zileuton (LB node in Community I – Immune system related drugs) is also effective in anticancer therapy [20][21].

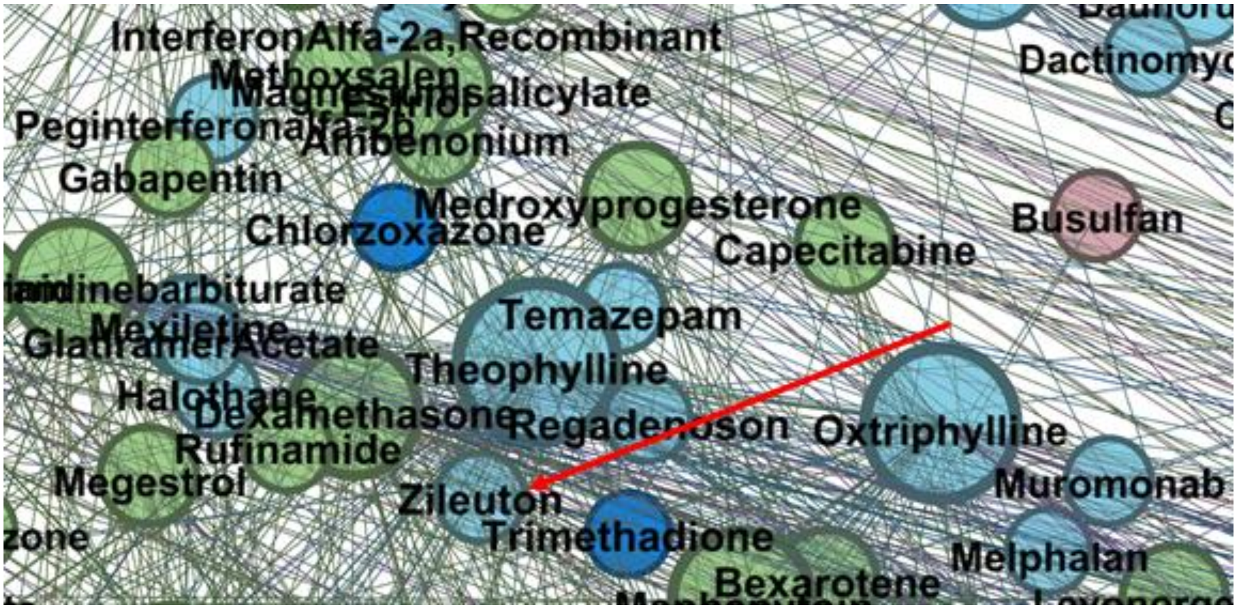

Figure 10. Zoomed zileuton in Community I (Immune system related drugs).

3.2.5 Erythromycin

Erythromycin is a VM (velvet maroon) node eccentrically placed in community II (CYP P450 acting drugs), because it is metabolized by CYP3A4, but at the border with community III (Nervous system acting drugs). This position suggests that erythromycin may have activity on nervous system (i.e. parasympathomimetic activity). It was recently reported that erythromycin, a motilin receptor agonist, acts as gastrokinetic agent via a cholinergic pathway [22][23].

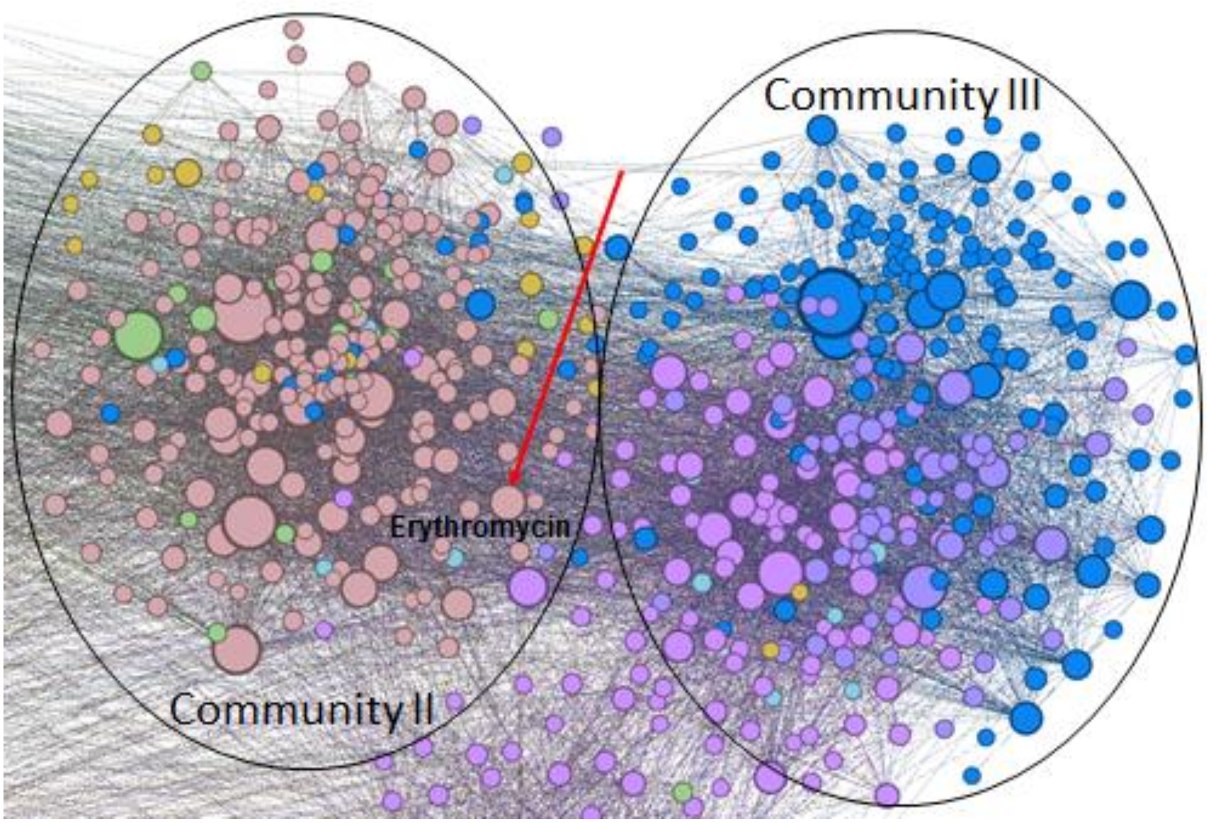

Figure 11. Zoomed erythromycin in Community II (CYP P450 acting drugs), at the border with Community III (Nervous system acting drugs).

.

3.2.6 Medroxyprogesterone and megestrol

Our methodology places antineoplastic endocrine drugs in Community IX (Epilepsy related drugs), but in the overlapping zone with Community I (Immune system related drugs). For example, medroxyprogesterone and megestrol are green nodes in Community IX. Indeed, medroxyprogesterone can prevent women's catamenial epilepsy [24][25], and megestrol has neuroprotective and anticonvulsant properties [26]. Medroxyprogesterone and megestrol are well-known hormonal antineoplastic agents[4].

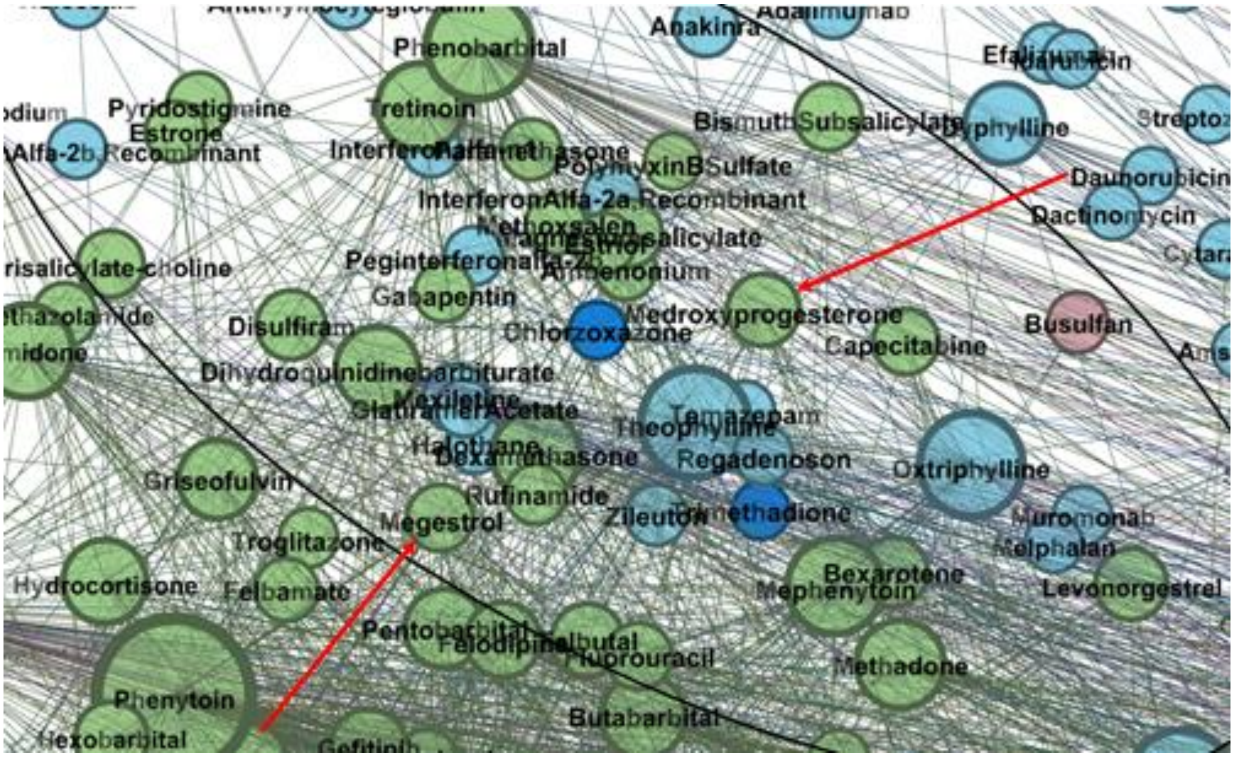

Figure 12. Zoomed detail with medroxyprogesterone and megestrol positioning in Community IX (Epilepsy related drugs), but in the overlapping zone with Community I (Immune system related drugs).

[4] Wishart DS, Knox C, Guo AC, Shrivastava S, Hassanali M, Stothard P, Chang Z, Woolsey J. DrugBank: a comprehensive resource for in silico drug discovery and exploration. Nucleic Acids Res. 2006 Jan 1;34(Database issue):D668-72 [http://www.drugbank.ca/]

3.2.7 *Disulfiram*

Our dual clustering methodology also reveals a general relationship between cancer and epilepsy, a conclusion that is also supported by recent research results in repositioning disulfiram as an anticancer drug [27][28][29]. Indeed, in our community-based drug-drug interaction network CBDDIN, disulfiram is a green node in Community IX (green modularity class generally characterizes Community IX), because it can induce seizures in the absence of ethanol challenge [30][31]; however, disulfiram lays in the overlapping zone with Community I – Immune system related drugs (Community I is mostly characterized by the light blue modularity class, which is associated with drugs targeting cancer, autoimmune disorders, and musculoskeletal system).

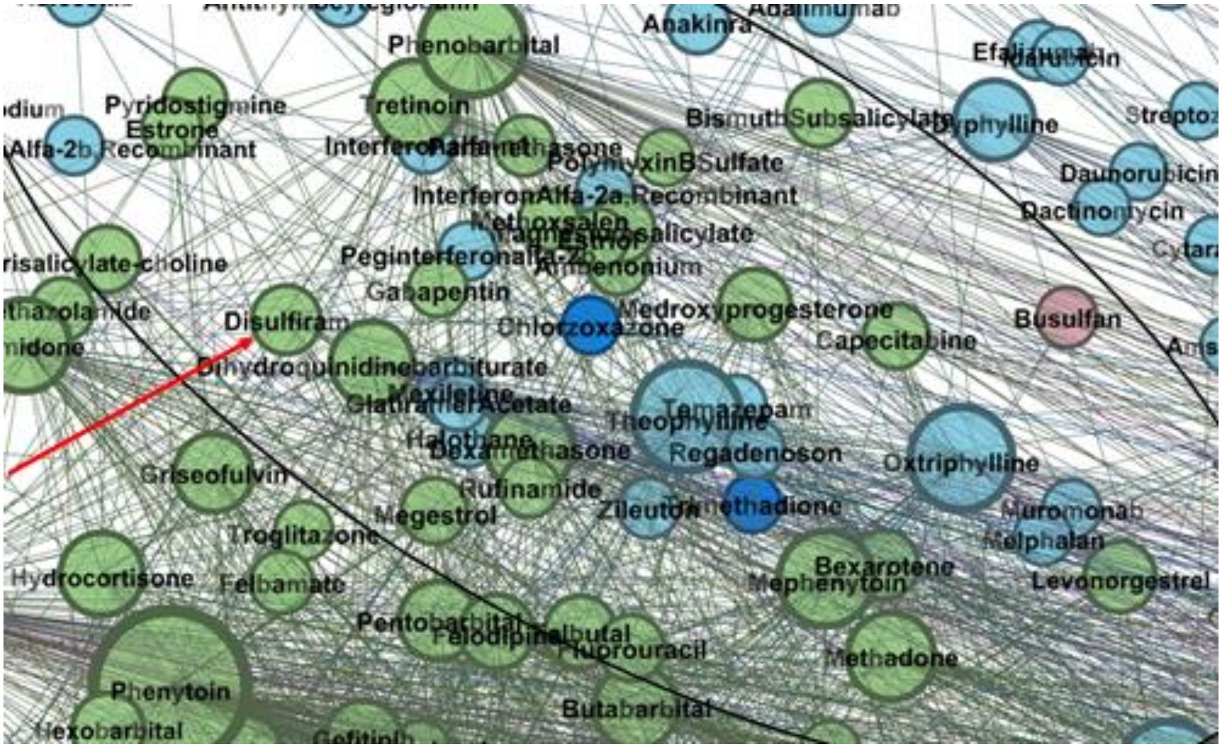

Figure 13. Zoomed disulfiram placement in the overlapping area of Communities I (Immune system acting drugs) and IX (Epilepsy related drugs).

3.2.8 Nifedipine

Nifedipine (green node) is a calcium channels blocker which is placed in Community IX (Epilepsy related drugs), due to the fact that it reduces seizure frequency when administered as adjuvant to antiepileptic treatment [32][33].

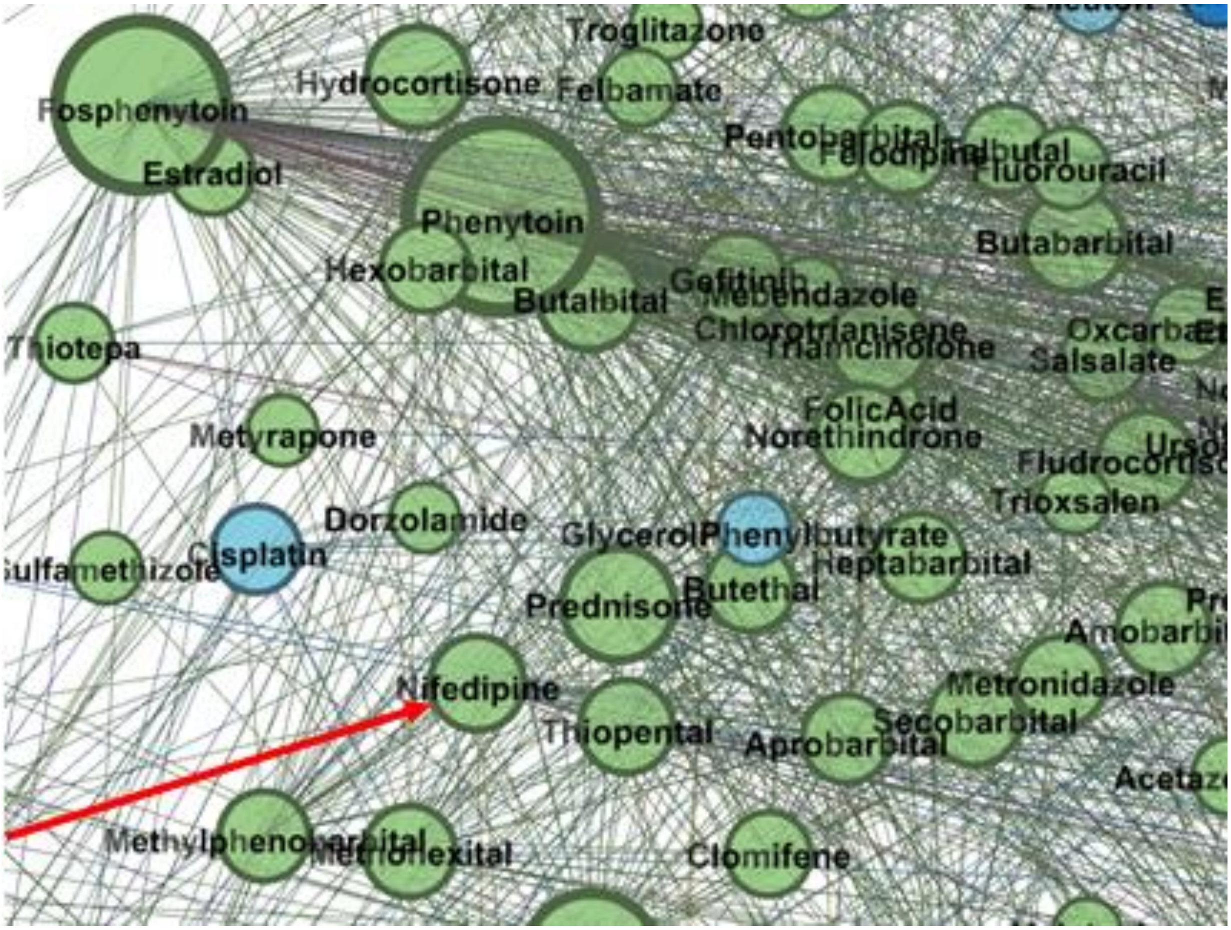

Figure 14. Zoomed nifedipine placement within the Community IX (Epilepsy related drugs).

3.3 Proposed properties

3.3.1 Topology-based predicted properties

Table 3. New drug properties derived from topological placement.

Drug	Current indication	Predicted property	Placement suggesting new property
Chlorzoxazone	Centrally acting central muscle relaxant	Anticancer	**Community I:** Immune system related drugs
Trimethadione	Antiepileptic		
Nizatidine	H_2 receptor antagonist	CYP P450 substrate/inhibitor/ inducer	**Community II:** Cytochrome P450 acting drugs
Troleandomycin	Antibiotic	Activity on nervous system	**Community III:** Nervous system acting drugs
Perflutren	Contrast media		
Montelukast	Anti-asthmatic drug	Sympathomimetic/ Sympatholytic	**Community IV:** Sympathetic nervous system acting drugs
Repaglinide	Oral hypoglycemic drug		
Ibuprofen	Nonsteroidal anti-inflammatory drug		
Triamterene	Potassium-sparing diuretics	Interference with platelet activity	**Community V:** Kalemia and platelet activity related drugs
Spironolactone			
Cyclophosphamide	Antineoplastic	Anti/Pro-hemorrhagic	**Community VI:** Hemostasis related drugs
Levamisole	Anthelmintic		
Amphotericin B	Antimycotic antibiotic	Muscle relaxant/myotonic	**Community VII:** Neuromuscular transmission acting drugs
Cefamandole	Antibiotic		
Methicillin	Antibiotic	Ligand	**Community VIII:** Metal cations complexes
Isotretinoin	Anti-acne retinoid		
Sulfamethizole	Antiinfective sulfonamide	Anti/Pro-epileptic	**Community IX:** Epilepsy related drugs
Melatonin	Psycholeptic		

3.3.2 Modularity-based predicted properties

Table 4. New drug properties derived from modularity classes.

Drug	Current indication	New property	Modularity suggesting new property
Penciclovir	Antiviral	Cancer/ autoimmune/ musculoskeletal disorders	**LB**: Drugs targeting cancer, autoimmune disorders (i.e. rheumatoid arthritis), and musculoskeletal system
Loracarbef	Antibiotic		
Telavancin	Antibiotic	Activity on nervous system	**DB**: Central and peripheral nervous system acting drugs
Bepridil	Anti-angina		
Ganciclovir	Antiviral	CYP enzymes substrate/inhibitor/inducer	**VM**: Substrates, inhibitors and inducers of specific CYP enzymes
Tolazoline	Peripheral vasodilator		
Griseofulvin	Antifungal antibiotic	Anti/Pro-Hemorrhagic, Anti/Pro-Convulsant	**G**: Drugs interfering in different phases of hemostasis, anticonvulsant and epileptogenic drugs
Orlistat	Anti-obesity		
Terbinafine	Antifungal	Sympathomimetic/ Sympatholytic	**M**: Sympathetic nervous system acting drugs
Bezafibrate	Hypolipidemic		
Nilutamide	Antineoplastic	Platelet aggregation inhibitor or inducer/kalemia level	**GB**: Drugs interfering with platelet activity and plasma potassium level
Liraglutide	Hypoglycemic		

References

[1] K. L. Johnson-Davis, G. A. McMillin, J. M. Juenke, C. D. Ford, F. B. Petersen. Which dose of busulfan is best? *Clin. Chem.* 56(7):1061–1064, 2010.

[2] M. Hirohashi, K. Takasuna, Y. Kasai, C. Usui, H. Kojima. Pharmacological studies with the alpha 2-adrenoceptor antagonist midaglizole. Part II: Central and peripheral nervous systems. *Arzneimittelforschung.* 41(1):19–24, 1991.

[3] K. C. Lee, D. C. Randall. Potentiation of the pressor response to stress by tolbutamide in dogs. *Integr. Physiol. Behav. Sci.* 28(1):22–28, 1993.

[4] M. A. Valentovic, W. C. Lubawy. Impact of insulin or tolbutamide treatment on 14C-arachidonic acid conversion to prostacyclin and/or thromboxane in lungs, aortas, and platelets of streptozotocin-induced diabetic rats. *Diabetes* 32(9):846–851, 1983.

[5] K. Kawaguchi, Y. Oribe, H. Uzawa. Tolbutamide effect on cultured human endothelial cells with special reference to platelet aggregation. *Tohoku J. Exp. Med.* 141(Suppl):563–568, 1983.

[6] V. Mašek, E. Anzenbacherová, M. Machová, V. Brabec, P. Anzenbacher. Interaction of antitumor platinum complexes with human liver microsomal cytochromes P450. *Anticancer. Drugs* 20(5):305–311, 2009.

[7] N. Raje, K. Anderson. Thalidomide - a revival story. *N. Engl. J. Med.* 341(21):1606–1609, 1999.

[8] M. T. Miller, K. Strömland. Teratogen update: thalidomide: a review, with a focus on ocular findings and new potential uses. *Teratology* 60(5):306–321, 1999.

[9] R. J. D'Amato, M. S. Loughnan, E. Flynn, J. Folkman. Thalidomide is an inhibitor of angiogenesis. *Proc. Natl. Acad. Sci.* 91(9):4082–4085, 1994.

[10] R. L. Barnhill, N. J. Doll, L. E. Millikan, R. C. Hastings. Studies on the anti-inflammatory properties of thalidomide: effects on polymorphonuclear leukocytes and monocytes. *J. Am. Acad. Dermatol.* 11(5):814–819, 1984.

[11] S. K. Teo, D. I. Stirling, J. B. Zeldis. Thalidomide as a novel therapeutic agent: new uses for an old product. *Drug Discov. Today* 10(2):107–114, 2005.

[12] T. Erkinjuntti, A. Kurz, S. Gauthier, R. Bullock, S. Lilienfeld, C. V. Damaraju. Efficacy of galantamine in probable vascular dementia and Alzheimer's disease combined with cerebrovascular disease: a randomised trial. *Lancet* 359(9314):1283–1290, 2002.

[13] H. A. M. Mucke. Drug repositioning: extracting added value from prior R&D investments. *Insight Pharma Reports*, 2010.

[14] G. K. Wilcock, S. Lilienfeld, E. Gaens. Efficacy and safety of galantamine in patients with mild to moderate Alzheimer's disease: multicentre randomised controlled trial. *BMJ* 321(7274):1445, 2000.

[15] K. Ito, S. Lim, G. Caramori, B. Cosio, *et al*. A molecular mechanism of action of theophylline: induction of histone deacetylase activity to decrease inflammatory gene expression. *Proc. Natl. Acad. Sci.* 99(13):8921–8926, 2002.

[17] L. Hirsh, A. Dantes, B.-S. Suh, Y. Yoshida, *et al*. Phosphodiesterase inhibitors as anti-cancer drugs. *Biochem. Pharmacol.* 68(6):981–988, 2004.

[18] G. Wang, V. Bhoopalan, D. Wang, L. Wang, X. Xu. The effect of caffeine on cisplatin-induced apoptosis of lung cancer cells. *Exp. Hematol. Oncol.* 4(1):1, 2015.

[19] E. A. Rogozin, K. W. Lee, N. J. Kang, H. Yu, *et al*. Inhibitory effects of caffeine analogues on neoplastic transformation: structure-activity relationship. *Carcinogenesis* 29(6):1228–1234, 2008.

[20] Z. Meng, R. Cao, Z. Yang, T. Liu, Y. Wang, X. Wang. Inhibitor of 5-lipoxygenase, zileuton, suppresses prostate cancer metastasis by upregulating E-cadherin and paxillin. *Urology* 82(6):1452–e7, 2013.

[21] X. Chen, S. Sood, C. S. Yang, N. Li, Z. Sun. Five-lipoxygenase pathway of arachidonic acid metabolism in carcinogenesis and cancer chemoprevention. *Curr. Cancer Drug Targets* 6(7):613–622, 2006.

[22] E. Deloose, R. Vos, P. Janssen, O. den Bergh, L. Van Oudenhove, I. Depoortere, J. Tack. The motilin receptor agonist erythromycin stimulates hunger and food intake through a cholinergic pathway. *Am. J. Clin. Nutr.* 103(3):730–737, 2016.

[23] J. Janssens, T. L. Peeters, G. Vantrappen, J. Tack, *et al*. Improvement of gastric emptying in diabetic gastroparesis by erythromycin: preliminary studies. *N. Engl. J. Med.* 322(15):1028–1031, 1990.

[24] A. G. Herzog. Catamenial epilepsy: definition, prevalence pathophysiology and treatment. *Seizure* 17(2):151–159, 2008.

[25] C. L. Harden, A. G. Herzog, B. G. Nikolov, B. S. Koppel, *et al*. Hormone replacement therapy in women with epilepsy: A randomized, double-blind, placebo-controlled study. *Epilepsia* 47(9):1447–1451, 2006.

[26] S. Baxendale, C. J. Holdsworth, P. L. M. Santoscoy, M. R. M. Harrison, *et al*. Identification of compounds with anti-convulsant properties in a zebrafish model of epileptic seizures. *Dis. Model. Mech.* 5(6):773–784, 2012.

[27] J. S. Shim, J. O. Liu. Recent advances in drug repositioning for the discovery of new anticancer drugs. *Int. J. Biol. Sci.* 10(7):654–663, 2014.

[28] D. Cen, R. I. Gonzalez, J. A. Buckmeier, R. S. Kahlon, N. B. Tohidian, F. L. Meyskens. Disulfiram induces apoptosis in human melanoma cells: a redox-related process1. *Mol. Cancer Ther.* 1(3):197–204, 2002.

[29] S. S. Brar, C. Grigg, K. S. Wilson, W. D. Holder, *et al*. Disulfiram inhibits activating transcription factor/cyclic AMP-responsive element binding protein and human melanoma growth in a metal-dependent manner in vitro, in mice and in a patient with metastatic disease. *Mol. Cancer Ther.* 3(9):1049–1060, 2004.

[30] R. D. McConchie, D. R. Panitz, S. R. Sauber, S. Shapiro. Disulfiram-induced de novo seizures in the absence of ethanol challenge. *J. Stud. Alcohol* 44(4):739–743, 1983.

[31] R. R. Kulkarni, B. K. Bairy. Disulfiram-induced de novo convulsions without alcohol challenge: Case series and review of literature. *Indian J. Psychol. Med.* 37(3):345, 2015.

[32] J. G. Larkin, E. Butler, M. J. Brodie, “Nifedipine for epilepsy? A pilot study. *Br. Med. J. (Clin. Res. Ed).* 296(6621):530, 1988.

[33] S. Otoom, Z. Hasan. Nifedipine inhibits picrotoxin-induced seizure activity: further evidence on the involvement of L-type calcium channel blockers in epilepsy. *Fundam. Clin. Pharmacol.* 20(2):115–119, 2006.
